# A Maltose-Binding Protein Fusion Construct Yields a Robust Crystallography Platform for MCL1

**DOI:** 10.1371/journal.pone.0125010

**Published:** 2015-04-24

**Authors:** Matthew C. Clifton, David M. Dranow, Alison Leed, Ben Fulroth, James W. Fairman, Jan Abendroth, Kateri A. Atkins, Ellen Wallace, Dazhong Fan, Guoping Xu, Z. J. Ni, Doug Daniels, John Van Drie, Guo Wei, Alex B. Burgin, Todd R. Golub, Brian K. Hubbard, Michael H. Serrano-Wu

**Affiliations:** 1 The Broad Institute, Cambridge, Massachusetts, United States of America; 2 Dana-Farber Cancer Institute and Howard Hughes Medical Institute, Boston, Massachusetts, United States of America; 3 Beryllium, Bedford, Massachusetts, United States of America; 4 Acme Bioscience, Palo Alto, California, United States of America; 5 Van Drie Research, North Andover, Massachusetts, United States of America; NCI-Frederick, UNITED STATES

## Abstract

Crystallization of a maltose-binding protein MCL1 fusion has yielded a robust crystallography platform that generated the first apo MCL1 crystal structure, as well as five ligand-bound structures. The ability to obtain fragment-bound structures advances structure-based drug design efforts that, despite considerable effort, had previously been intractable by crystallography. In the ligand-independent crystal form we identify inhibitor binding modes not observed in earlier crystallographic systems. This MBP-MCL1 construct dramatically improves the structural understanding of well-validated MCL1 ligands, and will likely catalyze the structure-based optimization of high affinity MCL1 inhibitors.

## Introduction

Evasion of programmed cell death, or apoptosis, is a hallmark of cancer that allows tumor cells to survive stresses that would kill a normal cell [1]. Specifically, cell death-inducing mitochondrial permeabilization is prevented by tight sequestration of membrane-localized proteins by anti-apoptotic members of the BCL-2 family, which include BCL-2, BCL-XL, BCL-W, A1, and MCL1 [2–3]. Human genetics points to a selective advantage of *MCL1*-amplified tumor cells. The analysis of over 3,000 diverse human tumors indicates that *MCL1* is among the top 10 most frequently amplified genes in human cancer [4–5]. Consistent with its frequent amplification, *MCL1* is highly expressed in many tumor types, and high expression levels of *MCL1* contribute to tumor development and resistance to chemotherapy [6–7].

There has been intensive effort to target anti-apoptotic members of the BCL-2 family with small molecules designed to release pro-apoptotic proteins from their sequestered state [8]. Both navitoclax, a dual inhibitor of BCL-XL and BCL-2, and ABT-199, a selective inhibitor of BCL-2, are currently in clinical investigation [9–10]. These small molecules effectively mimic one of the alpha helices, termed a BH3 helix, that pro-apoptotic proteins present to BCL-2 and or BCL-XL. The ability of these molecules to selectively target an expansive hydrophobic protein surface and disrupt high affinity protein-protein interactions is a remarkable achievement. Recently, other strategies to restore apoptosis via direct activation of two pro-apoptotic BCL-2 family members, BAX and BAK, have been described [11–12]. In both strategies, high-resolution structural data via NMR and X-ray crystallography were essential for ligand validation and subsequent optimization.

Molecular strategies to inhibit MCL1 have only recently emerged [13–18]. In total, only six MCL1-small molecule ligand structures have been deposited in the Protein Data Bank, compared to more than twenty for BCL-XL. That five of the six known MCL1-ligand structures display ligand/protein contacts both within and across adjacent crystallographic units strongly suggests that the crystallization of MCL1 protein has been highly ligand-dependent thus far. The absence of an apo MCL1 crystal structure underscores the high ligand dependence of existing crystallographic systems. Efforts to leverage structure-based design for MCL1 inhibitor optimization have certainly been hampered by the relative scarcity of structural insight.

In this report, we describe the development of a general and robust crystallography platform for soluble MCL1, using a combination of protein fusion and engineering strategies. This novel system has led to the first apo form of MCL1 characterized by X-ray crystallography, thus offering a powerful complement to the NMR apo MCL1 structure recently described [19]. We illustrate the utility of this MCL1 crystallography platform by solving the bound structure of several known MCL1 ligands, including low affinity fragments that had previously eluded structural characterization.

## Results

### Structure of MCL1 173–321 bound to Ligand 1

**Fig 1:**
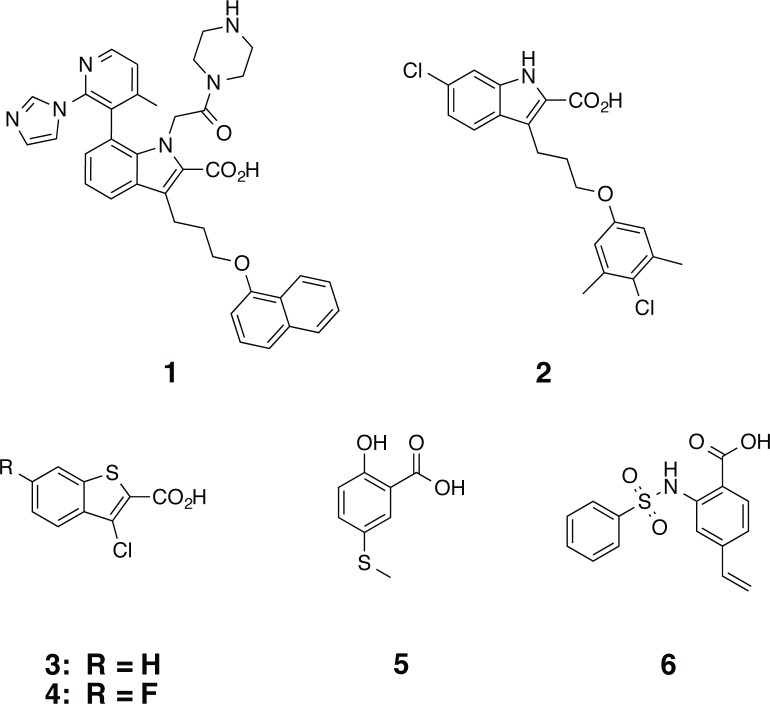
MCL1 ligands used in co-crystallization experiments.

Our initial efforts towards MCL1 ligand co-crystallization employed a truncated MCL1 protein similar to previously described constructs [15]. This construct, spanning residues 173–321, removed N-terminal regions that are predicted to have low structural organization as well as a C-terminal transmembrane domain. Using this construct, we embarked on an extensive co-crystallization screening campaign spanning structurally diverse ligands and broad matrix crystallization screens (Fig 1). Notably, we did not obtain crystals for apo MCL1 173–321, consistent with the apparent difficulty in obtaining a ligand-independent crystal form for MCL1.

**Fig 2:**
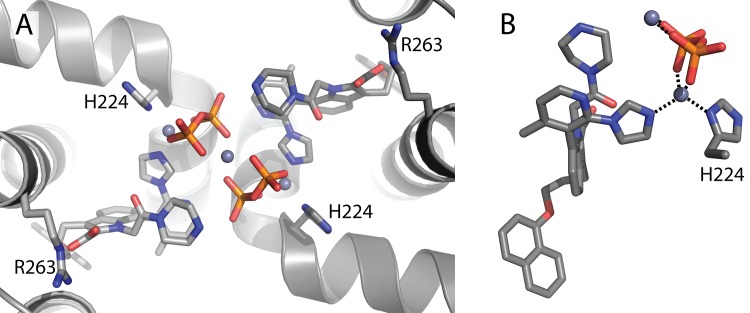
Crystal packing of MCL1 173–321 is mediated by zinc and pyrophosphate. (A) The structure of MCL1 173–321 was determined to 1.70 Å. (B) In the ligand-bound MCL1 173–321 structure, the imidazole group of **1** coordinates with zinc along with H224 and pyrophosphate.

Despite considerable effort, crystals were only identified for a single ligand, compound **1**, from a very specific crystallization condition containing 16% PEG8000, 20% glycerol, 40mM potassium phosphate, and 2mM zinc chloride. The structure of MCL1 173–321 was determined bound to **1** at 1.7 Å (Fig 2 and S1 Table). Interestingly, the naphthyl ether induces MCL1 sidechain shifts near M250 and F270 to reveal a small hydrophobic pocket. The carboxylic acid of the indole engages in two hydrogen bonds with R263, while the remaining portions of the ligand extend out and away from the main binding site of MCL1. One of the crucial crystal contacts in the structure was mediated by a bridging zinc ion that not only engages the imidazole of **1**, but also binds, through pyrophosphate, to a second zinc atom bound to a neighboring imidazole in the adjacent asymmetric unit. This highly unique crystal packing required the addition of Zn^2+^, as numerous crystallization trials with **1** but lacking Zn^2+^ (or other divalent metal ions) failed to produce crystals.

### Creation and crystallization of alternate MCL1 constructs

As the co-crystallization of MCL1 and ligand **1** would not be generally applicable to other ligands, we explored a protein engineering approach to develop a more robust MCL1 crystal system. Fusion of a target protein to a solubilizing partner is a frequent strategy for difficult proteins, where the partner protein can not only improve the expression and purification yield, but also introduce favorable crystal packing interactions [20]. N-terminal fusion constructs of MCL1 173–321 were created using thioredoxin (Trx), Lysozyme (Lyz), Sumo (Smt), and maltose binding protein (MBP), but soluble expression was only observed for Trx, Smt, and MBP. While we were aware of MBP point mutations that improve crystallization of other fusion proteins [21], our initial focus was on the wild-type MBP sequence.

Unfortunately, both the Trx-MCL1 and MBP-MCL1-WT proteins failed to produce crystals from broad matrix screens in the absence of small molecule ligands. Closer inspection of published MCL1 structures identified a highly disordered loop between residues G192 and S202. We therefore introduced three simultaneous alanine mutations (K194A, K197A, R201A) into the MBP-MCL1 construct, anticipating that these sidechain truncations would reduce overall surface entropy and possibly favor crystal packing. In parallel, two linkers were designed to reduce steric clash between the fused protein and MCL1: a short 2-residue linker (GS) and a longer 6-residue linker (GSGGGG) (S1 Fig). Both proteins were subjected to extensive broad matrix screening efforts in both the presence and absence of ligands, however crystals were only identified for the MCL1 K194A/K197A/R201A construct with the two-residue GS linker. We hereby refer to this construct bearing K194A/K197A/R201A and a two-residue GS linker as MBP-MCL1. Crystal quality was further improved by adding 2mM maltose to the protein prior to setting crystallization trials.

### MBP-MCL1 binds to peptides, ligands, and fragments in a similar affinity as MCL1 alone

The bioequivalence of our novel MBP-MCL1 fusion construct and wild-type MCL1 was established by affinity measurements of known MCL1 ligands using isothermal titration calorimetry (ITC). A comparison of binding affinities (Table 1 and S2 Fig) for the BH3 peptide Noxa (20–38), the small molecule BH3 mimetic **2**, and the fragment **6** confirms that the conformation of the MBP-MCL1 fusion protein is identical to the wild-type MCL1 protein, and that neither the point mutations in the disordered loop of MCL1 nor the fusion to MBP significantly perturbed the overall tertiary structure of MCL1.

**Table 1:** Binding affinity (K_D_) of ligands to MCL1 and MBP-MCL1.

Ligand	MCL1 173–329 (μM)	MBP-MCL1 (μM)
hNoxa (20–38)	0.160	0.190
**2**	0.060	0.180
**6**	18.0	14.1

All experiments are n ≥ 3, and averaged values for K_D_ are reported.

### Structure of Apo MBP-MCL1

**Fig 3:**
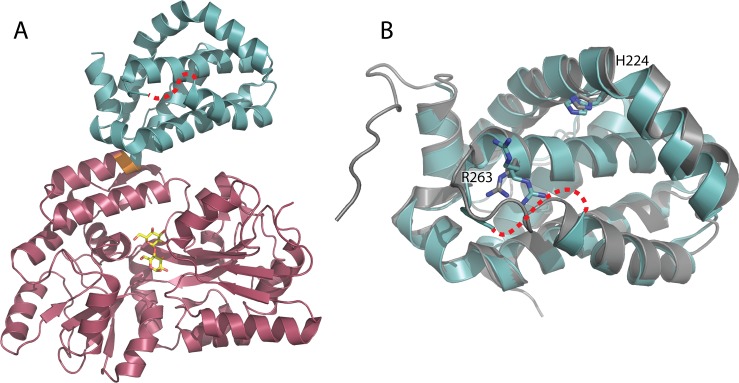
The structure of Apo MBP-MCL1 determined at 1.90 Å. (A) The MBP domain (red) is connected by a short GS linker (orange) to MCL1 173–321 (blue). A portion of alpha helix four is not ordered in the structure (red dashed-line). Maltose ligand is shown in yellow. (B) The MCL1 domain is structurally very similar to the NMR structure of Apo-MCL1 (gray).

The structure of MBP-MCL1 was determined in the apo state at 1.9 Å (Fig 3 and S1 Table). The presence of MCL1 does not change the conformation of MBP locally or globally (superposition r.m.s.d. of MBP-MCL1 and PDB ID 4MBP 0.47 Å overall, calculated on all common Cα). The Gly-Ser linker that connects MBP to MCL1 causes a 45° bend in the nearly continuous alpha helix between the MCL1 and MBP domains. The overall fold of MCL1 is similar to the recently reported apo NMR structure of MCL1 (PDB ID 2MHS) (superposition r.m.s.d. of MBP-MCL1 and PDB ID 2MHS 1.08 Å overall, calculated on all common Cα) and agrees well with the range of r.m.s.d. observed between other human MCL1 structures including peptide and ligand bound models in the PDB. The mean B-factor for the structure is low overall (~20 Å^2^), but increases to 40–50 Å^2^ starting at L235. The main chain is highly disordered between residues 253–257 (S3 Fig).

The loop containing three point mutations (K194A, K197A, and R201A) makes key packing interactions with symmetry-related MCL1 molecules as well as the MBP fusion protein. The K195A residue is within 3.2 Å of L75 from an MBP symmetry mate and R201A is 3.7 Å from K17 of the fusion MBP protein. Meanwhile, K197A faces towards the open solvent channel and is more than 6 Å away from the C-terminus of the neighboring MCL1 molecule. Taken together, these results imply that K197A and R201A drive the overall packing of this crystal form. This packing allows the BH3 binding groove to face away from MBP and towards a solvent channel, thus making this crystal form amenable to soaking or co-crystallization of fragments into MCL1.

Interestingly, the MBP fusion construct with a six-residue linker (GSGGGG) failed to crystallize despite extensive screening. Both MBP fusion constructs had similar thermal stability (MBP-MCL1 T_m_ = 55.7°C +/- 0.2; MBP-long-MCL1 T_m_ = 54.2°C +/- 0.4), providing evidence that the overall globular structure of these two proteins were comparable. The shorter GS linker permits a continuous alpha helix to join the N-terminus of MCL1 and the C-terminus of MBP, whereas the GSGGGG linker is predicted to interrupt secondary structure in this region.

### Structure of MBP-MCL1 bound to known ligands

**Fig 4:**
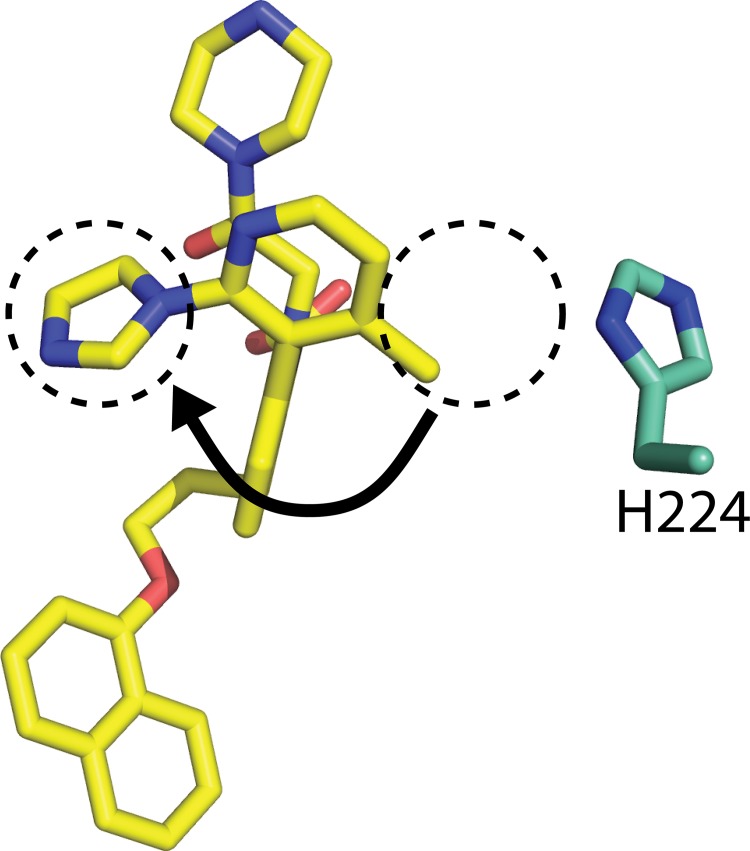
Removal of crystal packing interactions allows sidechain rotation of ligand 1. In the absence of zinc and pyrophosphate, the pyridine ring of **1** rotates 180° away from H224 allowing the imidazole to face out towards the solvent channel.

Most of the known ligand-bound structures of MCL1 crystallize through direct ligand-ligand interactions with adjacent proteins in the crystallographic unit cell, creating the possibility of false ligand orientations to mislead structure-based drug design. In our MBP-MCL1 crystals, the hydrophobic BH3 binding groove is largely solvent exposed, allowing this region to readily accommodate ligands without disrupting crystal packing interactions. As an initial proof of concept, we re-investigated the co-crystallization of ligand **1**, which in earlier efforts relied entirely on zinc-mediated interactions between ligand and neighboring protein. The structure of MBP-MCL1 was determined to 2.35 Å and has high overall similarity to the structure of MCL1 173–321 (superposition r.m.s.d. of MBP-MCL1 and MCL1 173–321 bound to **1**: 0.58 Å overall, calculated on all common Cα) (Fig 4).

Interestingly, in the absence of the zinc and pyrophosphate packing interactions, the pyridine ring rotates 180° from the previous non-MBP structure, with the 4-methyl group now pointing toward H224 and the imidazole group facing out towards solvent. This divergence provided the first evidence that our MBP-MCL1 fusion construct, by introducing novel crystal contacts distal to the ligand binding site, would allow ligand-independent crystallization of BH3-competitive ligands. A more important implication for structure-based design was the dramatic difference in ligand orientation between the MBP-MCL1 fusion and non-fused MCL1 structures.

**Fig 5:**
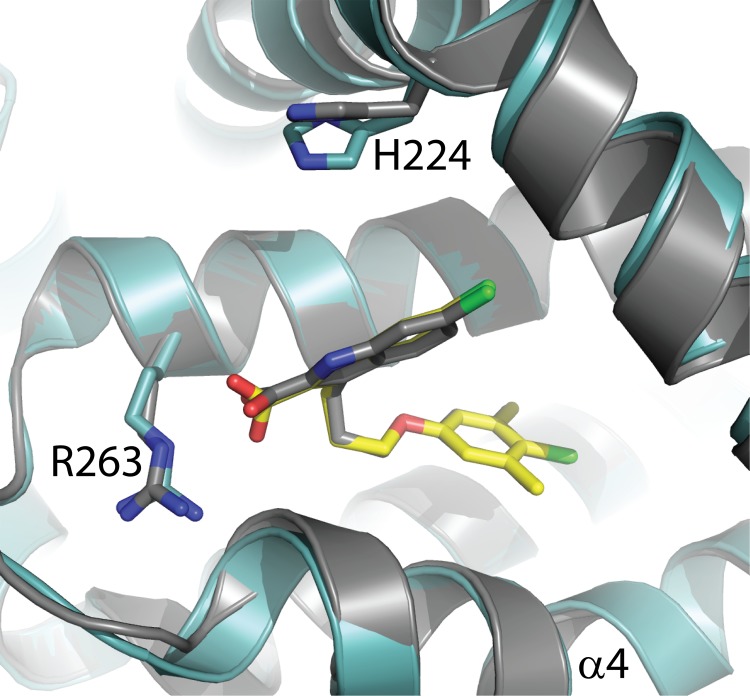
Comparison of PDB 4HW2 and MBP-MCL1 with ligand 2. The structure of MBP-MCL1 with ligand **2** (yellow) determined to 1.55 Å (blue) overlaid with the structure of MCL1 171–323 determined at 2.4 Å (PDB ID 4HW2, gray).

We next crystallized other known MCL1 ligands in our MBP-MCL1 system. Fesik and co-workers have recently described the indole carboxylate **2** and solved the structure bound to MCL1 (171–321) at 2.8 Å resolution (PDB ID 4HW2). In one of the packing interactions for 4HW2, R207 from a neighboring molecule creates a single hydrogen bond to the carboxylic acid of the ligand and makes close contact with a loop spanning residues 193–198. Ligand **2** readily co-crystallized in our MBP-MCL1 fusion construct, and the structure of this complex was solved at 1.55 Å (Fig 5). Overall, the structure of MBP-MCL1 bound to **2** replicates the structure of PDB ID 4HW2 (superposition r.m.s.d. of MBP-MCL1 bound to **2** and PDB ID 4HW2 0.48 Å overall, calculated on all common Cα).

### Low-affinity fragments co-crystallized with MCL1

Compound **2** was discovered via fragment-based screening methods, where a benzothiophene carboxylate **3** was merged with a second fragment to afford a higher-affinity compound. NMR-derived distance restraints were used to generate a computational pose of fragment **3** in the BH3 binding groove of MCL1 and the fragment was extended to create the inhibitor bound in PDB ID 4HW3. Independently, we identified a related fragment **4** using ^19^F-based NMR fragment screening. As before, the affinity of **4** was identical between the wild-type MCL1 protein and our MBP-MCL1 fusion construct (ITC K_D_ = 97 and 93 μM respectively).

**Fig 6:**
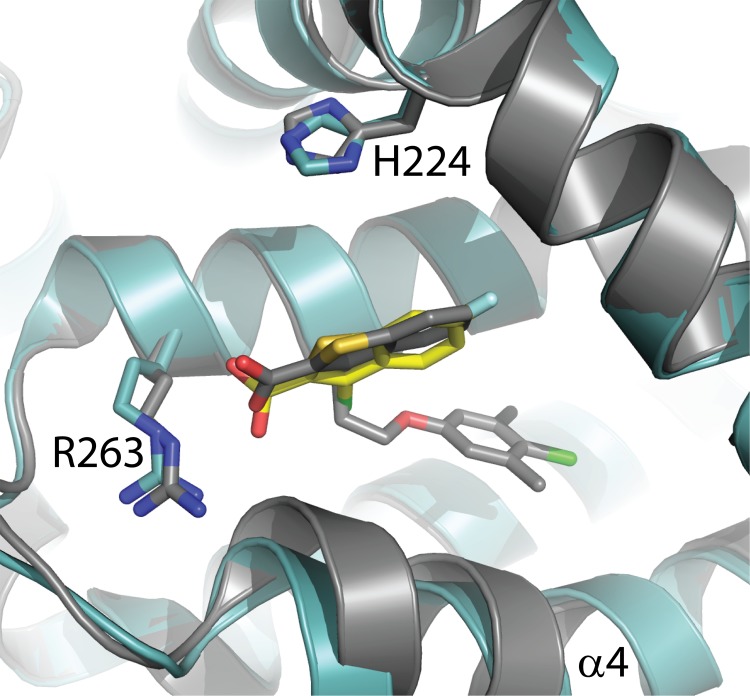
Comparison of PDB 4HW3 and MBP-MCL1 with fragment 4. The structure of MBP-MCL1 with fragment **4** (yellow) determined to 2.4 Å (blue) overlaid with the structure of MCL1 171–323 determined at 2.4 Å (PDB ID 4HW3, gray). The carboxylic acid of 4HW3 adopts multiple conformations depending on the chain; only chain A is shown for clarity.

Due to the accessibility of the BH3 binding pocket, we were able to soak fragment **4** into crystals of MBP-MCL1 and determine the MCL1-ligand structure to 2.4 Å (Fig 6). To our knowledge, this structure marks the first example of a low-affinity fragment co-crystallized with MCL1. MBP-MCL1 bound to fragment **4** adopts a very similar conformation to PDB ID 4HW3 (superposition r.m.s.d. of MBP-MCL1 bound to 4 and PDB ID 4HW3 0.48 Å overall, calculated on all common Cα). With the absence of the propyl ether extension, the end of alpha helix 4 (residues 250–255) shifts away from the ligand binding site. As expected, the benzothiophene carboxylate engages R263, and the heterocyclic core overlays well with the indole ring of **2**. Interestingly, the carboxylic acid of **4** is rotated 50° relative to that of **2**, allowing the formation of a second hydrogen bond to R263 when compared to the previous structure.

Additional fragment hits that bind MCL1 have also been described by Petros and co-workers [18]. Using the N- and C-terminally truncated MCL1 construct described previously, they were unable to crystallize any of the fragment hits. Instead, only the higher-affinity derivatives were amenable to crystallography (PDB ID 4OQ5 and 4OQ6). Leveraging our MBP-MCL1 construct, we were able to soak fragments into apo MBP-MCL1 crystals, namely the salicylate **5** and the vinyl sulfonamide **6** (Fig 1).

**Fig 7:**
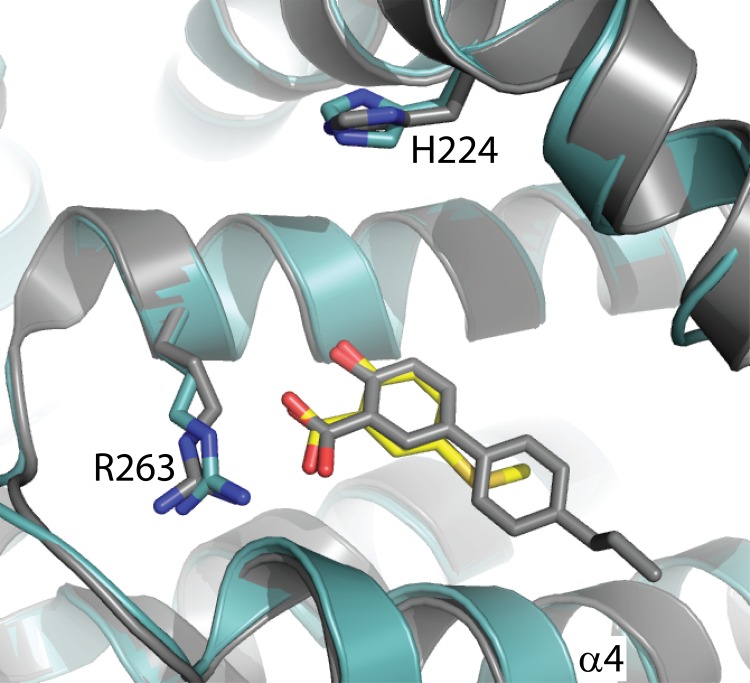
The structure of MBP-MCL1 bound to fragment 5 determined at 1.9 Å. Fragment **5** (yellow) binds similarly in comparison to the elaborated ligand from PDB ID 4OQ6 (gray).

The structure of MBP-MCL1 was determined bound to fragment **5** at 1.9 Å (Fig 7). For this ligand, the bound orientations of the fragment and biphenyl derivative PDB ID 4OQ6 are nearly identical (superposition r.m.s.d. of MBP-MCL1 bound to **5** and PDB ID 4OQ6 0.36 Å overall, calculated on all common Cα). However, in our MBP-MCL1 structure with fragment **5**, R263 of MCL1 adjusts to create two hydrogen bonds with ligand. Meanwhile, the ortho-phenol of **5** forms an intramolecular hydrogen bond in addition to a third hydrogen bond with the peptide backbone of L267. R263 in PDB ID 4OQ6 is shifted away and makes only a single hydrogen bond to ligand. In the elaborated salicylate of 4OQ6, no hydrogen bond between ligand and L267 is evident.

**Fig 8:**
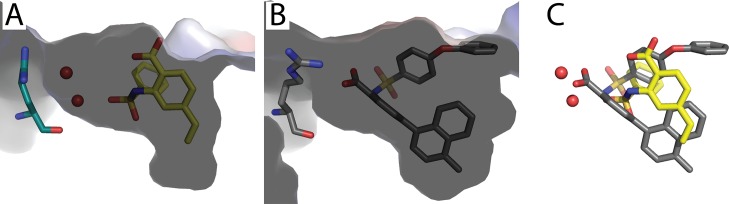
Structure of MBP-MCL1 bound to fragment 6 determined at 2.0 Å. (A) The surface side-view shows that fragment **6** shifts and makes water mediated hydrogen bond contacts with the peptide backbone of R263. (B) The elaborated ligand of fragment **6** (PDB ID 4OQ5) shifts to allow the methyl-naphthalene to bind in the hydrophobic pocket, requiring the carboxylic acid to make a single hydrogen bond with the sidechain of R263. (C) Overlay of crystallized fragment **6** and the elaborated ligand in PDB ID 4OQ5 reveals a distinct binding pose for **6**.

The structure of MBP-MCL1 was determined bound to fragment **6** at 2.0 Å and reveals a dramatically different ligand conformation in comparison to the elaborated derivative from PDB ID 4OQ5 (Fig 8). The overall protein structures are highly similar, but significant differences are observed in the ligand binding site (superposition r.m.s.d. of MBP-MCL1 bound to fragment **6** and PDB ID 4OQ5 0.49 Å overall, calculated on all common Cα). In contrast to our other fragment structures and PDB ID 4OQ5, the carboxylic acid from **6** does not make any hydrogen bonds with the guanidinium sidechain of R263, but instead forms a water-mediated interaction with the backbone amide carbonyl (Fig 8A). Meanwhile, the 4-vinyl substituent reaches towards M250 and F270, and the phenyl ring of **6** is positioned within a hydrophobic cleft created by A227, M231, and F270. The planar core of **6** is almost perpendicular to that of 4OQ5 and tilts the vinyl substituent down into the pocket near M250 and F270 by ~30° (Fig 8C). Within a chemical series, a shift in ligand disposition is not surprising, as additional (hydrophobic) interactions can alter orientation. Earlier structural knowledge of how fragment **6** bound to MCL1, however, may have encouraged a medicinal chemistry path distinct from previous efforts.

## Discussion

In this report, we introduce an MBP-fusion strategy to afford the first high-resolution apo structure of MCL1 solved by X-ray crystallography as well as a robust platform for obtaining fragment and ligand bound structures of MCL1. The success of this fusion strategy rests on at least two cooperating factors: 1) reduction in surface entropy by targeted mutagenesis of disordered loop residues, and 2) maintenance of secondary alpha-helical structure in the linker region between the MBP and MCL1 proteins. Importantly, neither of these protein engineering strategies compromises the function and fidelity of the MCL1 protein structure, thus supporting the use of structural insights from this MBP-MCL1 construct for inhibitor design.

There are more than 60 unique structures that have been reported using the MBP-fusion strategy. In most of these structures, the C-terminal alpha helix of MBP is followed by an unstructured linker before secondary structure (of the target protein) resumes. Only six of these structures contain a continuous alpha helix encompassing the C-terminus of MBP and the N-terminus of the fused protein (PDB IDs 4IRL, 4FEB, 2VGQ, 3N94, 3VD8, 3WAI). Additionally, the MBP fusion used in this study was based on the wild-type sequence, while many of the MBP fusion structures are based on specific point mutations on MBP to induce crystal packing. When looking at all MBP-fusion structures, the linker region of the MBP-adenylate cyclase receptor fusion construct (PDB ID 3N94) most closely resembles the 45° bend observed in the MBP-MCL1 structure. While we currently do not understand the importance of this continuous alpha-helix in favoring crystallization of MCL1, our inability to crystallize the MBP-MCL1 construct bearing a six-residue linker underscores the importance of linker variation in engineering fusion protein constructs.

In this MBP-MCL1 fusion construct, the key protein-protein interface that MCL1 uses to sequester BH3 partner proteins is largely solvent exposed and is not involved in crystal contacts. Instead, key packing interactions are observed in the loop region where three alanine point mutants were introduced, namely residues K194A, K197A, and R201A. The steric accessibility of this hydrophobic groove is a key advantage of this MBP-MCL1 construct in studying the bound state of BH3-competitive ligands. Crystallization of these ligands in a ligand-independent manner has dramatically increased the opportunities for structure-based optimization of diverse inhibitor chemotypes. Conversely, the strong ligand-dependency of previously reported MCL1 crystallization systems likely explains the low number of MCL1 ligand structures despite extensive efforts in the field.

In addition to providing a more versatile system for MCL1 ligand co-crystallization, this MBP-MCL1 construct has enabled the crystallization of low-affinity fragments that had previously eluded structural characterization despite significant effort by us and others. Fragment-based methods are an important hit generation strategy, especially for challenging protein-protein interactions where traditional biochemical methods are not available. Highly sensitive biophysical methods are available to detect ligands that bind at millimolar affinity, thus necessitating a complementary structural toolbox to unequivocally validate binding and inform fragment optimization.

We have also demonstrated multiple examples where the bound conformation of known ligands in our MBP-MCL1 fusion construct diverges from previously-determined crystal structures. In our own hands, removal of ligand- and zinc-mediated crystal packing revealed a new sidechain orientation where the imidazole and methyl substituents were opposite to the non-MBP structure. In the case of the sulfonamide ligand **6**, successful crystallization of the parent fragment revealed an entirely different pose relative to the elaborated ligand. Each of these examples underscores the potential risk of using ligand-dependent crystallization systems for structure-based design.

**Fig 9:**
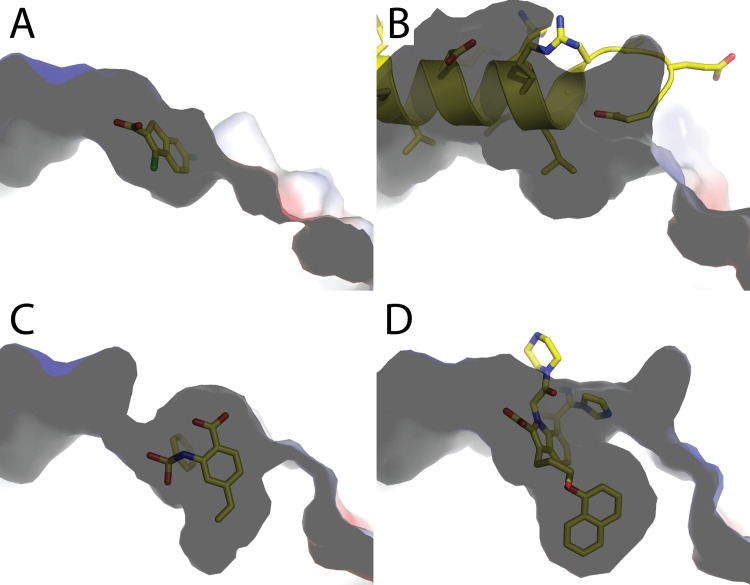
The conformational flexibility of the binding pocket of MCL1. Surface representations are shown as side views and ligands are shown as yellow sticks. (A and B) Fragment 4 maps onto L78 of NoxaB from PDB ID 2NLA, with only minor structural perturbation of the BH3-binding groove of MCL1. In contrast, binding of fragment 6 creates a significant pocket (C) which is further expanded upon binding of ligand 1 (D).

In aggregate, the structural characterization of diverse MCL1 ligands spanning peptides, fragments, and potent small molecule BH3 mimetics allows a detailed understanding of the conformational dynamics of MCL1. With a fragment such as benzothiophene **4**, the single interaction of the ligand carboxylate with R263 of MCL1 leads to greater structural order with minor backbone shifts in alpha helix 4 relative to the BH3 peptide-bound state (Fig 9A and 9B). Synthetic fragments such as **6**, however, capture conformational states not seen in previous peptide or small molecule structures (Fig 9C). Upon binding of fragment **6**, three residues (L246, M250, and F254) shift to create hydrophobic surfaces that accommodate binding of non-polar fragments (Fig 9C). This hydrophobic interaction is further exploited by higher affinity ligands such as **1** that shifts the sidechains of L267 and F254 to create an even larger hydrophobic pocket (Fig 9D). The necessary sidechain movement to create a hydrophobic “hole” in the BH3-binding groove of MCL1 is not easily anticipated from the apo structure. The breathability of MCL1 is further suggested by the disorder of residues 253–257 in α-helix four of apo MBP-MCL1. One might speculate that this conformational flexibility is necessary for MCL1 and other anti-apoptotic BCL-2 family members to effectively bind the diverse group of BH3-containing proteins known to promote apoptosis. In fact, the dynamic interplay between conformational states of MCL1 and the BH3 motif from PUMA further has recently been studied by stopped flow kinetics and molecular dynamics [22]. This study underscores that even structurally well-defined proteins such as MCL1 should not be viewed as static templates when binding protein partners.

Within the BCL-2 family, clinical development of MCL1-specific inhibitors has lagged behind investigational therapies targeting BCL-XL and BCL-2. A number of factors, including the suggestion of on-target toxicity observed with MCL1 genetic ablation [23–24], may have hindered the development of safe and effective MCL1 therapeutics thus far. Design of a highly potent small molecule to displace MCL1 from its high affinity protein partners is a necessary step in MCL1 therapeutic efforts. Additional structural information on inhibitor binding and the successful translation of direct binding to more complex *in vivo* systems should catalyze further progress towards more potent MCL1 inhibitors.

## Materials and Methods

### Molecular biology

The MCL1 gene inserts for all constructs were based on nucleotide accession number AK316267, and were all flanked with unique 5’ BamHI and 3’ HindIII sites, with bases “TGAT” just preceding the HindIII site so as to introduce two stop codons prior to the HindIII site. Amino acid numbering for MCL1 is based on the amino acid sequence from NCBI accession number AAF64255. MCL1 173 – 329 was amplified with the appropriate restriction sites (KOD, Novagen), then subcloned via BamHI/HindIII into vector pEMB54, resulting in an arabinose-inducible construct expressing 6XHis-Smt3-MCL1 173 – 329. This MCL1 insert was truncated by PCR (KOD, Novagen) to produce MCL1 173 – 321, which was then subcloned via BamHI/HindIII into vector pEMB128, resulting in N-term 6XHis-Tev-MBP (maltose binding protein)-MCL1 173 – 321 (IPTG-inducible). Additional MBP-MCL1 constructs were created by mutagenesis (Quikchange, Agilent) to generate MCL1 173 – 321 containing the mutations K194A, K197A, and R201A; this insert was then subcloned by BamHI/HindIII into vector pEMB124 to afford N-term 6XHis-Tev-Trx (thioredoxin)-MCL1 173 – 321 K194A, K197A, R201A. The same insert was subcloned into pEMB128 to afford N-term 6XHis-Tev-MBP (maltose binding protein)-MCL1 173 – 321 K194A, K197A, R201A (IPTG-inducible). The 6XHis-MBP portion of the ORF from N-term 6XHis-Tev-MBP (maltose binding protein)-MCL1 173 – 321 K194A, K197A, R201A was modified to include a 4 glycine linker at the C-term of MBP, flanked with NcoI/BamHI and subcloned into NcoI/BamHI-digested vector.

The pEMB54 vector was created by making the following modifications to pBAD22: The BamHI site just upstream of the BAD promoter was mutated from GGATCC to GGCTCC, then a 6XHis-Smt3 insert was cloned into the EcoRI/HindIII sites of the modified pBAD22 multiple cloning site. The insert included a unique BamHI site just after the Smt3 gene and before the HindIII site, allowing subsequent inserts to be cloned in-frame to N-term 6XHis-Smt3 by BamHI/HindIII restriction-based cloning. The pEMB124 vector was created by subcloning an N-term 6XHis-Tev-Trx insert via EcoRI/HindIII into vector pEMB54, with a unique BamHI site just after the Trx sequence, allowing subsequent inserts to be cloned via BamHI/HindIII in-frame with this tag. The pEMB128 vector was created by subcloning an N-term 6XHis-Tev-MBP insert via NcoI/HindIII into pET28a (Novagen), with a unique BamHI site just after the MBP sequence, allowing subsequent inserts to be cloned via BamHI/HindIII in-frame with this tag.

### Fermentation of MCL1, MBP-MCL1 Fusion, TRX-MCL1 Fusion and Mutants

Briefly, the target specific vector was transformed into either Top 10 or BL21 (DE3) *Escherichia coli* cells. A starter culture containing either 100 μg/mL (final concentration) of ampicillin or 50 μg/mL (final concentration) kanamycin (Teknova) was inoculated with a single colony and grown 16 hours at 37°C. This was then transferred to 8 liters of terrific broth (Teknova) containing the appropriate antibiotic as described above, which was grown to OD_600_ = 0.6. Protein expression was induced by adding either 0.1% arabinose or 1mM isopropyl-beta-D-thiogalactopyranoside (IPTG) (VWR) as appropriate and the cultures were grown at either 25°C or 30°C overnight or at 37°C for three hours. The cells were harvested by centrifugation (Beckman) at 5000 rpm for 15 minutes and the pellets were collected and stored at –80°C.

### Protein Purification of MCL1, MBP-MCL1 Fusion, TRX-MCL1 Fusion and Mutants

Cells were resuspended in 50mM HEPES pH 7.5, 500mM NaCl, 5% glycerol, 0.001% 3-[(3-Cholamidopropyl)dimethylammonio]-1-propanesulfonate (CHAPS) (JT Baker), 0.5mM Tris-(2-carboxyethyl)phosphine (TCEP) (VWR), 250 U Benzonase (Novagen), 100 mg lysozyme (Sigma) and one complete EDTA free protease inhibitor tablet (Roche), lysed via sonication and clarified via centrifugation. The lysate was filtered with a bottle top filter. The supernatant was applied to a Ni^2+^ charged HiTrap Chelating HP (GE Healthcare) column and the protein eluted with 20 mM HEPES pH 7.5, 500 mM NaCl, and with either 2mM or 5mM TCEP or no reducing agent present over a 500mM imidazole gradient. The affinity tag (either His-Smt or His-TEV) was removed via cleavage with either Ubiquitin-like-specific protease 1 (Ulp-1) or Tobacco Etch Virus Protease (TEV) during dialysis. The affinity tag was captured through reverse Ni^2+^ chromatography. The flow through contained the cleaved protein. The protein was concentrated via centrifugal concentration and further purified via size exclusion chromatography over either a Sephacryl S-100 16/60 or 26/60 (GE Healthcare) column in 20mM HEPES pH 7.5, 200mM NaCl, 1% or 5% glycerol and either 2mM DTT, 5mM TCEP or no reducing agent present. The fractions containing the target protein were pooled and concentrated for crystallography via centrifugal concentration to 10 mg/mL.

### Chemistry

Ligands 1–2, 4, and 6 were synthesized or purchased per below:

- Ligand 1: Synthesized according to WO2008130970 (Abbott); CAS #1073067-90-9
- Ligand 2: Synthesized as described [15]; CAS #1415968-47-6
- Ligand 4: Purchased from TimTec; CAS #34576-92-6
- Ligand 5: Synthesized; CAS #32318-42-6
- Ligand 6: Synthesized; CAS #681241-71-4

### Biophysics

Isothermal titration calorimetry (ITC) experiments were conducted with each ligand in order to determine binding affinities for each of the MCL1 crystallization constructs. Solutions of 250μM of ligand **2** or 800μM of fragment **4** or **6** were prepared in Buffer A (25mM Hepes, pH 7.4, 100mM NaCl, 0.1mM TCEP, 4% DMSO) for titration into each protein (MCL1 or MBP-MCL1) at 25μM in dialysis-matched Buffer A. Titrations were conducted using a MicroCal Auto-ITC200 instrument (Malvern Instruments, Ltd.) at 25°C, with a stirring speed of 1000 rpm and a set reference power of 10 μcal/sec. As a control experiment, each compound was titrated into Buffer A under the same conditions and compound heat of dilution effects were subtracted from the titrations into protein. Titration curves were analyzed using Origin software version 7.0552, provided by MicroCal, and values for K_D_ were calculated for each compound binding to each protein construct.

### Crystallization

MCL1 173–321 at 10 mg/mL was incubated with 1mM ligand **1** and 2mM zinc chloride for 10 minutes at 16°C prior to setting up sitting drop crystallization trials. Incubated protein was mixed in equal ratios with 16% PEG8000, 20% glycerol, and 40mM potassium phosphate at 16°C. Crystals appeared within 4 days. Crystals were flash frozen using liquid nitrogen. Crystallization trials were set with MBP-MCL1 (10 mg/mL) in the presence of 2mM maltose in the presence or absence of ligand. The protein solution was incubated for 10 minutes at 16°C and sitting drop crystallization trials were set by mixing equal portions of protein and mother liquor (19–25% (w/v) PEG-3350, 50-170mM Magnesium Formate) at 16°C. Crystals typically appeared after 24–48 hours. Ligands were dissolved in 100% DMSO and were added to the co-crystallization trials at concentrations ranging from 0.5 – 2 mM. Apo and co-crystals were cryo protected using a quick soak in 20% (w/v) PEG-3350, 104.4 mM Magnesium Formate, 12% Ethylene Glycol, and 0.8 mM maltose. Fragments were soaked in using the same cryo protection solution with the addition of 0.5 – 2 mM of the fragment for two days. All crystals were flash frozen using liquid nitrogen.

### X-ray data collection and processing

All datasets were collected at 100 K on a Rigaku FR-E+ SuperBright rotating anode generator with VariMax optics and a Saturn 944 detector, at the Advanced Photon Source beamline 21-ID-F with a Marmosaic 225 CCD detector, or at the Canadian Macromolcular Crystallization Facility beamline 08ID-1 with a Marmosaic 225 CCD detector. All data were reduced with XDS/XSCALE [25]. The structure of MCL1 173–321 was phased by molecular replacement using Phaser from the CCP4 suite of programs with PDB ID 4OQ6 used as a search model [26–27]. Apo MBP-MCL1 was phased by molecular replacement using Phaser from the CCP4 suite of programs with MCL1 173–321 structure and 4MBP as search models. Subsequent structures of MBP-MCL1 were solved by utilizing the phases from the apo MBP-MCL1 structure and performing rigid body refinement in REFMAC [28]. All structures were completed using multiple cycles of refinement in Phenix followed by manual rebuilding of the structure using Coot [29–30]. All structures were quality checked by Molprobity [31]. Some of the data reported was supported by the SBGrid software platform [32]. All data reduction and refinement statistics are shown in S1 Table and have been deposited in the PDB (PDB IDs: 4WMR, 4WMS, 4WMT, 4WMU, 4WMV, 4WMW, 4WMX). Fig.s, overlays, and electrostatic surface potentials were created using Pymol (The PyMOL Molecular Graphics System, Version 1.5.0.4 Schrödinger, LLC.)

## Supporting Information

S1 Fig SDS-PAGE of MCL1 constructs used in crystallographic studies. Samples were reduced and loaded at 6μg; lane 2: MCL1 173–321, lane 3: MBP-MCL1 lane 4: MBP-MCL1-GSGGGG, lane 5: MBP-MCL1-WT.
(DOCX)

S2 Fig Isothermal titration calorimetry (ITC) data. Representative ITC titration curves for MCL1 173–329 and MBP-MCL1. All experiments were repeated (n ≥ 3), and averaged values for K_D_ were reported in Table 1. (A) 400 μM hNoxa (20–38), (B) 250 μM compound **2**, and (C) 800 μM compound **6** titrated into 25 μM MCL1-173-329. (A) 300 μM hNoxa (20–38), (B) 250 μM compound **2**, and (C) 800 μM compound **6** titrated into 25 μM MBP-MCL1. All experiments were performed with an autoITC200 instrument, at 25°C, in buffer composed of 25mM Hepes, pH 7.4, 100 mM NaCl, 0.1 mM TCEP and 4% DMSO. In experiments (C) and (F), the stoichiometry was set to 1 so that K_D_ and ΔH could be calculated.
(DOCX)

S3 Fig The structure of Apo MCL1. Multiple structures of Apo MCL1 were solved however the end portion of alpha helix 4 was always absent. Individual alpha helicies are shown as 173–191 blue, 202–224 cyan, 225–235 green, 240–253 red, 260–281 orange, 284–302 gray, 303–308 yellow, 311–319 pink.
(DOCX)

S1 Table X-ray data processing and refinement statistics. (DOCX)
