# Supplementary material for: A Maltose-Binding Protein Fusion Construct Yields a Robust Crystallography Platform for MCL1
Source: PLoS One. 2015 Apr 24;10(4):e0125010. doi: 10.1371/journal.pone.0125010 (PMC4409056; doi:10.1371/journal.pone.0125010)
Supplement: S1 Fig — Samples were reduced and loaded at 6μg; lane 2: MCL1 173–321, lane 3: MBP-MCL1 lane 4: MBP-MCL1-GSGGGG, lane 5: MBP-MCL1-WT. (DOCX) [file pone.0125010.s001.docx]

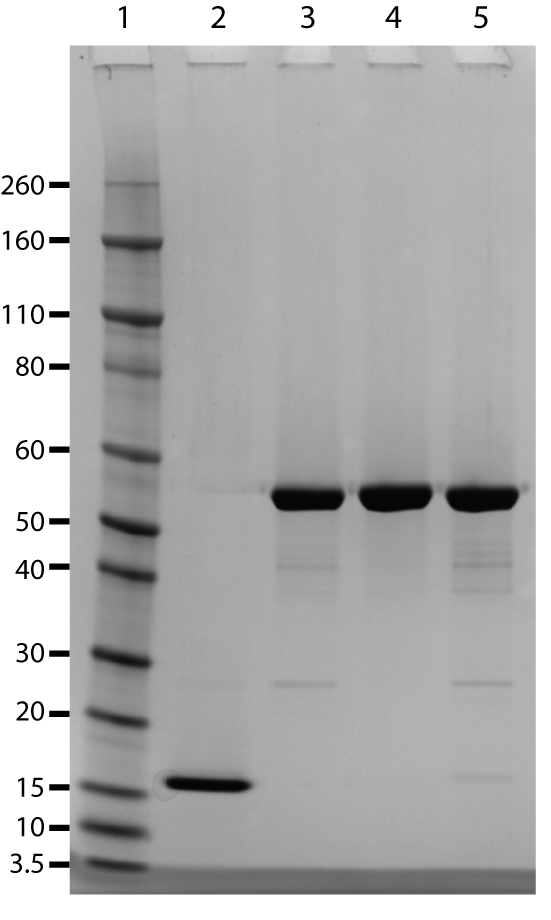


**Figure S2**: **SDS-PAGE of MCL1 constructs used in crystallographic studies.** Samples were reduced and loaded at 6µg; lane 2: MCL1 173-321, lane 3: MBP-MCL1 lane 4: MBP-MCL1-GSGGGG, lane 5: MBP-MCL1-WT.
