# Supplementary material for: A Maltose-Binding Protein Fusion Construct Yields a Robust Crystallography Platform for MCL1
Source: PLoS One. 2015 Apr 24;10(4):e0125010. doi: 10.1371/journal.pone.0125010 (PMC4409056; doi:10.1371/journal.pone.0125010)
Supplement: S2 Fig — Representative ITC titration curves for MCL1 173–329 and MBP-MCL1. All experiments were repeated (n ≥ 3), and averaged values for KD were reported in Table 1. (A) 400 μM hNoxa (20–38), (B) 250 μM compound 2, and (C) 800 μM compound 6 titrated into 25 μM MCL1-173-329. (A) 300 μM hNoxa (20–38), (B) 250 μM compound 2, and (C) 800 μM compound 6 titrated into 25 μM MBP-MCL1. All experiments were performed with an autoITC200 instrument, at 25°C, in buffer composed of 25mM Hepes, pH 7.4, 100 mM NaCl, 0.1 mM TCEP and 4% DMSO. In experiments (C) and (F), the stoichiometry was set to 1 so that KD and ΔH could be calculated. (DOCX) [file pone.0125010.s002.docx]

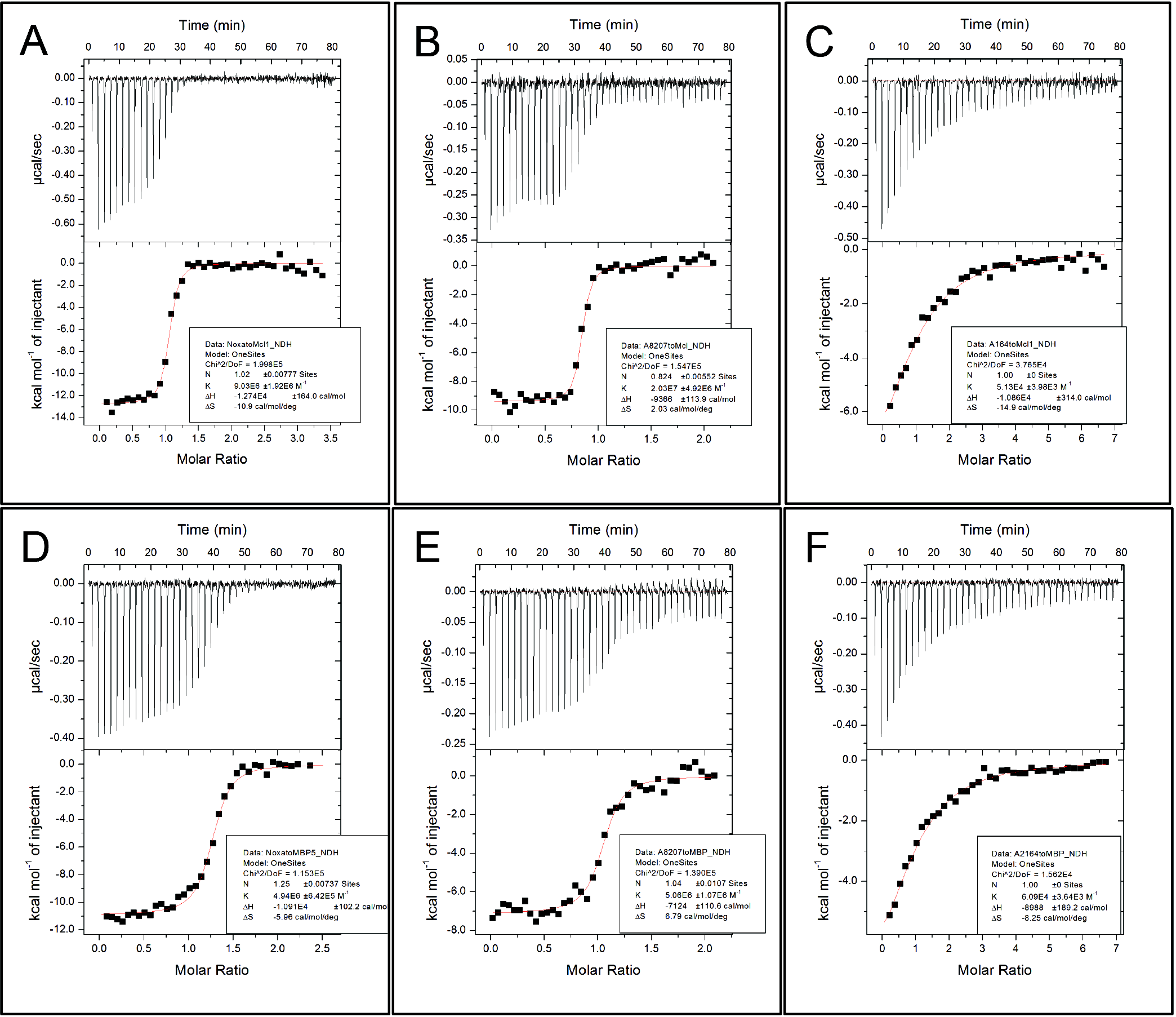


**Figure S3**: **Isothermal titration calorimetry (ITC) data.** Representative ITC titration curves for MCL1 173-329 and MBP-MCL1. All experiments were repeated (n ≥ 3), and averaged values for K_D_ were reported in Table 1. (A) 400μM hNoxa (20-38), (B) 250μM compound **2**, and (C) 800μM compound **6** titrated into 25μM MCL1-173-329. (A) 300μM hNoxa (20-38), (B) 250μM compound **2**, and (C) 800μM compound **6** titrated into 25μM MBP-MCL1. All experiments were performed with an autoITC200 instrument, at 25°C, in buffer composed of 25mM Hepes, pH 7.4, 100mM NaCl, 0.1mM TCEP and 4% DMSO. In experiments (C) and (F), the stoichiometry was set to 1 so that K_D_ and ΔH could be calculated.
