# Supplementary material for: A Maltose-Binding Protein Fusion Construct Yields a Robust Crystallography Platform for MCL1
Source: PLoS One. 2015 Apr 24;10(4):e0125010. doi: 10.1371/journal.pone.0125010 (PMC4409056; doi:10.1371/journal.pone.0125010)
Supplement: S3 Fig — Multiple structures of Apo MCL1 were solved however the end portion of alpha helix 4 was always absent. Individual alpha helicies are shown as 173–191 blue, 202–224 cyan, 225–235 green, 240–253 red, 260–281 orange, 284–302 gray, 303–308 yellow, 311–319 pink. (DOCX) [file pone.0125010.s003.docx]

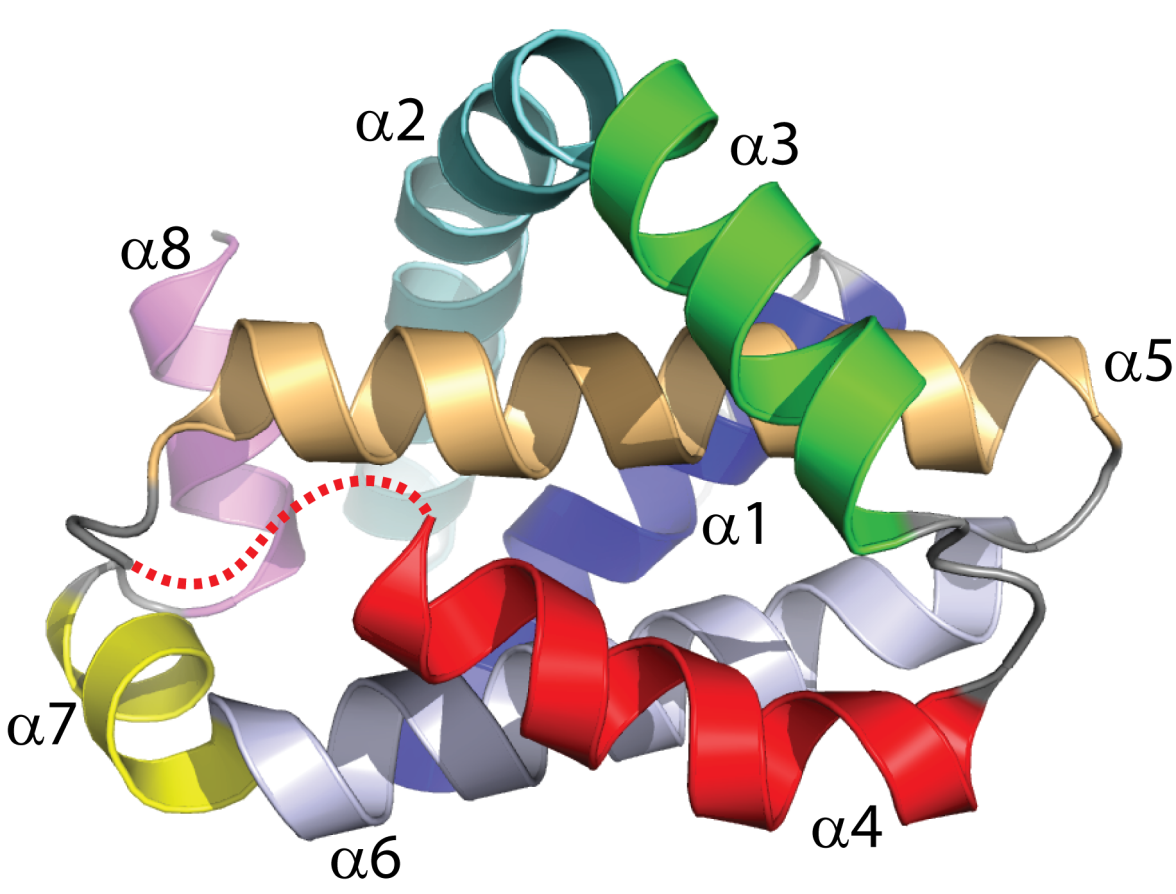


**Figure S4: The structure of Apo MCL1.** Multiple structures of Apo MCL1 were solved however the end portion of alpha helix 4 was always absent. Individual alpha helicies are shown as 173-191 blue, 202-224 cyan, 225-235 green, 240-253 red, 260-281 orange, 284-302 gray, 303-308 yellow, 311-319 pink.
