# Supplementary material for: A Maltose-Binding Protein Fusion Construct Yields a Robust Crystallography Platform for MCL1
Source: PLoS One. 2015 Apr 24;10(4):e0125010. doi: 10.1371/journal.pone.0125010 (PMC4409056; doi:10.1371/journal.pone.0125010)
Supplement: S1 Table — (DOCX) [file pone.0125010.s004.docx]

**Table S1**: **X-ray data processing and refinement statistics**

| Protein | MCL1 173-321 | MBP-MCL1 | MBP-MCL1 | MBP-MCL1 | MBP-MCL1 | MBP-MCL1 | MBP-MCL1 |
| --- | --- | --- | --- | --- | --- | --- | --- |
| Ligand | **1** | none | **1** | **2** | **4** | **5** | **6** |
| X-ray source | Rigaku FR-E+ | Rigaku FR-E+ | Rigaku FR-E+ | CLS 08id | Rigaku FR-E+ | Rigaku FR-E+ | Rigaku FR-E+ |
| a (Å) | 72.77 | 99.05 | 98.97 | 98.87 | 98.89 | 98.94 | 98.56 |
| b (Å) | 38.41 | 136.10 | 136.30 | 135.9 | 136.07 | 136.3 | 135.87 |
| c (Å) | 48.33 | 37.51 | 38.36 | 37.68 | 37.82 | 37.8 | 37.8 |
| α, β, γ | 90, 102.6, 90 | 90, 90, 90 | 90, 90, 90 | 90, 90, 90 | 90, 90, 90 | 90, 90, 90 | 90, 90, 90 |
| Space group | C2 | P2_1_2_1_2 | P2_1_2_1_2 | P2_1_2_1_2 | P2_1_2_1_2 | P2_1_2_1_2 | P2_1_2_1_2 |
| Wavelength (Å) | 1.5418 | 1.5418 | 1.5418 | 0.97949 | 1.5418 | 1.5418 | 1.5418 |
| Resolution limit (Å)* | 50-1.70 (1.74-1.70) | 50-1.90 (1.95-1.90) | 50-2.35 (2.41-2.35) | 50-1.55 (1.61-1.55) | 50-2.4 (2.49-2.40) | 50-1.9 (1.95-1.90) | 50-2.0 (2.05-2.0) |
| Number of observations* | 129144 (3335) | 538557 (26801) | 152843 (11023) | 455371 (43827) | 87913 (8658) | 248850 (18295) | 297756 (11669) |
| Completeness (%)* | 97.7 (84.8) | 98.2 (92.8) | 98.2 (84.9) | 100.0 (100.0) | 100.0 (99.0) | 99.8 (100.0) | 98.7 (86.7) |
| Multiplicity* | 9.7 (3.6) | 13.4 (9.7) | 6.9 (5.7) | 6.1 (6.0) | 4.2 (4.3) | 6.0 (6.2) | 8.4 (4.6) |
| Rmerge (%)* | 0.045 (0.291) | 0.067 (0.486) | 0.107 (0.389) | 0.061 (0.500) | 0.139 (0.506) | 0.052 (0.481) | 0.084 (0.579) |
| Mean I/σI * | 30.40 (4.18) | 27.93 (4.61) | 14.78 (4.76) | 17.91 (3.50) | 9.87 (3.27) | 20.99 (3.68) | 20.58 (3.2) |
| Refinement | | | | | | | |
| Resolution Range (Å) | 47.17-1.70 | 40.04-1.90 | 41.23-2.35 | 46.46-1.55 | 40.00-2.4 | 41.29-1.90 | 50-2.0 |
| Rcryst | 0.169 | 0.174 | 0.167 | 0.165 | 0.178 | 0.185 | 0.172 |
| Rfree | 0.206 | 0.214 | 0.215 | 0.193 | 0.253 | 0.216 | 0.217 |
| No. protein atoms | 1182 | 3916 | 3921 | 4005 | 3875 | 3900 | 3945 |
| No. ligand atoms | 47 | 62 | 107 | 107 | 39 | 34 | 44 |
| No. solvent atoms | 144 | 425 | 274 | 506 | 145 | 324 | 398 |
| Mean B-factor (Å) | 17.6 | 20.04 | 29.78 | 20.72 | 27.36 | 27.19 | 23.0 |
| Rmsd bond lengths (Å) | 0.006 | 0.007 | 0.002 | 0.006 | 0.005 | 0.004 | 0.007 |
| Rmsd bong angles (˚) | 1.42 | 0.99 | 0.62 | 1.02 | 0.81 | 0.80 | 0.97 |
| Ramachandran statistics (%) | | | | | | | |
| Favored | 97 | 99 | 98 | 99 | 97 | 98 | 99 |
| Allowed | 3 | 1 | 2 | 1 | 3 | 2 | 1 |
| Outlier | 0 | 0 | 0 | 0 | 0 | 0 | 0 |
| PDB ID | 4WMR | 4WMS | 4WMT | 4WMU | 4WMV | 4WMW | 4WMX |

* Highest resolution shell show in parentheses
